# An effective treatment in Erdheim Chester disease: vemurafenib: a case report

**DOI:** 10.1186/s13256-023-04153-z

**Published:** 2023-10-12

**Authors:** Ersin Bozan, Tahir Darçın, Samet Yaman, Tuğçe Nur Yiğenoğlu, Merih Kızıl Çakar, Mehmet Sinan Dal, Fevzi Altuntaş

**Affiliations:** Department of Hematology and Bone Marrow Transplantation Center, Hematology and Bone Marrow Transplantation Unit, Health Sciences University Ankara Dr. Abdurrahman Yurtaslan Oncology Training and Research Hospital, Ankara, Turkey

**Keywords:** Erdheim Chester disease, Vemurafenib, BRAF V600E, Mutation, Rare diseases

## Abstract

**Background:**

Erdheim Chester disease (ECD) is a rare disease with multisystemic involvement in the group of non-langerhans cell histiocytosis. Although nearly 100 years have passed since its definition, the number of cases reported all over the world is below 1000. In addition to the rarity of the disease, low awareness seems to play a role in this.

**Case presentation:**

47-year-old white caucasian women patient who presented to our clinic with symptoms of weakness-fatigue as well as increasing pain in the knees and ptosis in the left eye. Result of the patient's bone biopsy, ECD was considered pathologically and BRAF V600E mutation was shown molecularly. After presenting the clinical, laboratory and other examination results of the case, the dramatic response seen with targeted therapy will be discussed.

**Conclusions:**

BRAF V600E mutation is frequently seen in ECD. Vemurafenib plays an active role in targeted therapy.

## Background

Erdheim Chester disease (ECD) is a rare non-Langerhans cell histiocytic multisystem disease and is thought to arise from monocyte-macrophages, unlike Langerhans cell histiocytosis [1]. With the increase in the use of diagnostic imaging and laboratory tests, an increase is observed in the number of cases. The average age of occurrence is 50–60 years and the male to female ratio is 3 to 2. Child cases are very rare [2, 3].

ECD is a disease of myeloid progenitor cells. Mutations in BRAF V600E and other signaling pathways are thought to contribute to the process and play a role in the increase in inflammatory cytokines. No infectious, environmental, or hereditary etiology was found. It is thought to be due to acquired mutations [4, 5]. The BRAF V600E mutation has been demonstrated in approximately half of the cases. BRAF mutation activates the RAS/RAF/MEK/MAPK signaling pathway, increasing cell proliferation and inhibiting apoptosis. BRAF is a proto-oncogene encoded at codon 600 of exon 15, which encodes cytoplasmic serine/threonine kinase and activates the mitogen-activated protein kinase signaling pathway. Hairy cell mutation can be observed in leukemia, malignant melanoma, colorectal cancer, papillary thyroid cancer and histiocytosis. In the BRAF V600E mutation, E (glutamic acid) is coded instead of V (valine). Negatively charged glutamic acid creates an endogenous phosphorylation effect and causes uncontrolled activation of the gene [6, 7].

The clinic presentation differs according to the location and number of the affected area. Mostly bone involvement and at least 1 extra area involvement are seen. Some patients are asymptomatic and can be found incidentally in imaging studies, as well as patients with multisystemic involvement and aggressive course. Bone pain, neurological findings, diabetes insipidus, and constitutional symptoms such as weakness-fatigue may be observed [2].

## Case presentation

A 47-year-old white caucasian women patient applied to the orthopedics clinic with progressively worsening pain in both knees. The patient had no known chronic disease and no history of surgery. There was no known genetic disease history in the patient's family or herself. Osteosclerotic areas were seen in the diaphysis and metaphyses of both knees in the direct radiograph. She was sent to our clinic after the biopsy from the distal right femur revealed CD68 and CD163 positive, S100 and CD1a negative staining histiocytes. ECD disease consequently was determined to be the primary diagnosis.

In her clinical evaluation, the patient had tenderness in both knee joints and had ptosis in the left eye. Right shoulder movement was limited and painful. Laboratory and imaging tests of the patient were performed. No significant pathology was detected in laboratory tests, biochemistry and complete blood count tests. On cranial MR imaging, soft tissue obliterating all paranasal sinuses and extending to the left orbital superior wall was observed. Echocardiographic evaluation revealed minimal pericardial effusion and a chiari network in the right atrium. Intense involvement was observed in the whole body bone scintigraphy, especially in both nasomaxillary regions and both knee joints. The patient had limited range of motion and pain in the right shoulder, and a finding in favor of minimal effusion and tendinitis was found in shoulder MRI.

The biopsy specimen was evaluated by an another pathologist, confirming the diagnosis of ECD, and genetic analysis showed the BRAF V600E mutation.

Vemurafenib was started as a targeted therapy for the patient with BRAF V600E mutation. In the first month of the treatment, complaints of rash on the scalp and hair loss occurred. After the treatment was interrupted, the treatment was started again when the complaints regressed.

**Fig. 1:**
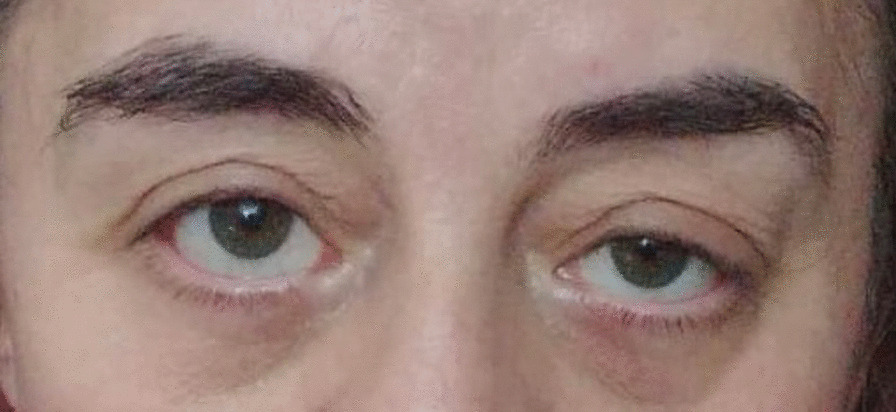
Before treatment

**Fig. 2:**
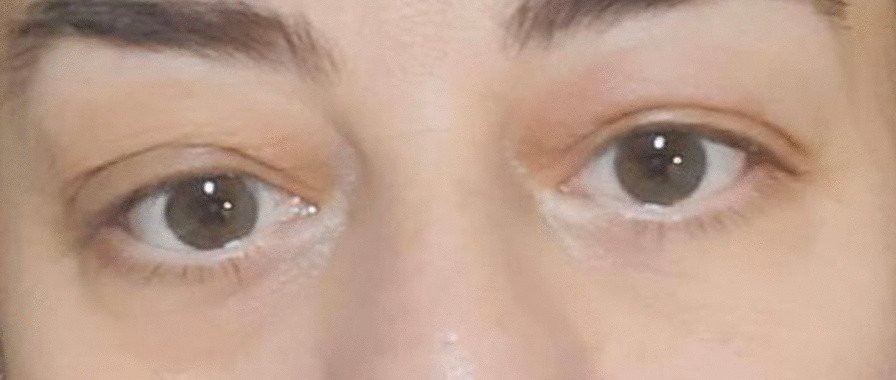
After treatment

In the evaluation made at the 3rd month of the treatment, it was observed that the ptosis in the left eye regressed (Fig. 1—before treatment, Fig. 2—after treatment) and the mobility of the right shoulder improved. At the 3rd month of the treatment control imaging, significant regression was observed in the soft tissue obliterating the sinuses in the cranial MRI. Pericardial effusion was not observed in the control cardiac evaluation. The patient is currently being followed up and treated in our clinic with vemurafenib treatment.

## Conclusions

ECD is a multisystemic disease in the non-langerhans cell histiocytosis group, with less than 1000 cases reported since its definition [8]. The clinic can be very different because it changes depending on the site of involvement [3].

Involvements in which osteosclerotic lesions are observed in the diaphysis and metaphyseal regions of the lower extremities are present in almost all patients. Bone and joint pain is the most common symptom seen in patients [9].

Cardiac involvement frequently plays a role in morbidity and mortality. Right heart involvement is most common. Periaortic fibrosis, periarterial coronary artery infiltration, pericardial thickening and effusion are other signs of cardiac involvement. Thoracic and abdominal 'covered aorta' may be seen due to circumferential involvement of soft tissue [10, 11].

Coronary artery involvement may cause infarction and renal artery involvement may cause hypertension [11, 12].

Central nervous system (CNS) involvement is observed in nearly half of the patients [13]. CNS involvement, whether symptomatic or not, is an independent risk factor for poor prognosis [14]. There can be many different types of involvement, but the most common complication of neurodegenerative cerebellar disease is observed. Exophthalmos, oculomotor palsy, and blindness can be seen due to unilateral or bilateral eye involvement. Thirst, frequent urination, cerebral palsy, loss of libido, headache, ataxia, dysarthria, convulsions, spinal cord compression may be observed depending on the site of involvement [15, 16].

Pulmonary involvement is observed in approximately 25–50% of the patients. Involvement of the pleura, parenchyma or both is observed. Although it may be asymptomatic, dyspnea and/or cough are observed [17].

Pathologically, they express CD14 (lipopolysaccharide receptor), CD68 (lysosomal macrosialin), CD163 (hemoglobin, haptoglobulin degradation receptor), factor XIIIa (tissue glutaminase). They do not express CD1a or CD207 (langerin), one of the Langerhans cell markers, and S100 positivity is rarely seen [18, 19].

Dramatic response is observed with vemurafenib treatment, which is effective in BRAF V600E mutation. We observed a similar effect in our patient.

The frequency of infection is increasing due to soft tissue involvement. The presence of infection and chronic disease can worsen the prognosis of the condition [20, 21].

Through this patient, we wanted to emphasize that possible morbidity and mortality in ECD, which can have multisystemic and vital organ involvement, can be prevented by early diagnosis and targeted treatment.

## Data Availability

Not applicable.
